# Sexually Dimorphic Association of Catechol-O-Methyltransferase (COMT) Functional Polymorphism with Dimensional Personality Pathology: A Preliminary Study

**DOI:** 10.3390/brainsci15121337

**Published:** 2025-12-16

**Authors:** John T. Rucker, Bishal Lamichhane, William B. Haren, Benjamin L. Weinstein, Alok Madan

**Affiliations:** 1 The Austen Riggs Center, Stockbridge, MA 01262, USA; johnruckerpsyd@gmail.com; 2 Menninger Department of Psychiatry and Behavioral Health, Baylor College of Medicine, Houston, TX 77030, USA; 3 Department of Psychiatry & Behavioral Health, Houston Methodist, Houston, TX 77030, USA; blamichhane@houstonmethodist.org (B.L.);; 4 Department of Psychiatry, Weill Cornell Medical College, New York, NY 10065, USA

**Keywords:** COMT, personality disorders, dimensional personality measures, sexual dimorphism

## Abstract

**Objective:** Genetic and environmental factors influence the expression of personality pathology and subsequent treatment efforts. This study associates genetics with a contemporary nosology of personality pathology represented in the Alternative Model for Personality Disorders (AMPD). We hypothesized traits from Criterion B of the AMPD would differ between genotypes of the catechol-O-methyltransferase (COMT) polymorphism (rs4680/Val158Met variation), given this genetic marker’s role in the metabolism of dopamine and norepinephrine, especially in the prefrontal cortex. **Methods:** The Personality Inventory for DSM-V (PID-5) was used to quantify personality traits, and the Genomind platform was used to identify the genotypes of the Val158Met COMT polymorphism in 84 psychiatric outpatients. **Results:** One of the five Criterion B personality domains and three of the twenty-five traits were significantly different among genotypes. Met/Met carriers had significantly higher pathological scores on the broad domain of negative affect and specific traits of perceptual dysregulation and separation insecurity, while the Val/Val carriers had significantly higher scores on the restricted affectivity trait. The COMT Val158Met polymorphism’s association with personality pathology was sexually dimorphic, with the two domains and nine traits significantly different across genotypes in males, but no differences were found in females. A substantial improvement in the regression of domains/traits score when gene–sex interactions were included further confirmed the dimorphism, e.g., the R-squared (adjusted) for the psychoticism improved from 0.03 (*p* = 0.15) to 0.19 (*p* < 0.001). **Conclusions:** Findings offer preliminary support for a link, potentially dimorphic across sexes, between the COMT Val158Met polymorphism and personality pathology as represented by the AMPD.

## 1. Introduction

Personality pathology represents global and longstanding imbalances in psychological functioning originating from genetic and environmental liabilities. In that personality pathology challenges traditional treatment approaches [1,2], understanding genetic influences contributing to the development and expression of personality pathology may be an essential step towards efficacious treatment strategies. One genetic marker with an emerging empirical connection to personality pathology is the dopamine-centric catechol-O-methyltransferase (COMT; [3,4]). The most commonly observed rs4680/Val158Met single-nucleotide polymorphism (SNP) of COMT has three genotypes: Valine/Valine (Val/Val), Valine/Methionine (Val/Met), and Methionine/Methionine (Met/Met), representing high, intermediate, and low COMT enzyme activity, respectively [5]. The COMT enzyme degrades catecholamine neurotransmitters such as dopamine and norepinephrine, contributing to variation in neural processes relevant to psychopathology, e.g., by modulating dopaminergic signaling in the prefrontal cortex. A review by Montag et al. [4] identified a link between COMT polymorphisms, negative emotionality, and anxiety disorders; however, they cautioned against oversimplifying the genetic salience of COMT polymorphisms. In a sample of relatives of patients diagnosed with schizophrenia and related disorders, Silberschmidt and Sponheim [6] found that groups with the Val allele had lower scores on personality traits of narcissism, rejection sensitivity, and stimulus-seeking than those with a Met allele. Zhang et al. [7] observed the COMT polymorphism moderated the relation between childhood abuse and psychopathy, with Val/Val carriers demonstrating higher rates of psychopathy as relates to proactive aggression. More recently, Mitrovic et al. [8] reported that the Met allele was linked with traits of self-consciousness, social anxiety, and social inhibition. In contrast, the Val allele was associated with higher extraversion, achievement striving, gregariousness, warmth, and positive emotions. These recent observations somewhat contrast Calati et al. [3], who found traits of aggressiveness and anger were higher for Val-dominant subjects. A meta-analysis by Taylor [9] investigating the specific Val158Met polymorphism of COMT reported pleiotropic effect of the polymorphism across multiple psychiatric disorders, further highlighting COMT’s relevance in understanding predispositions for transdiagnostic mental health ailments.

A substantial literature has examined COMT polymorphisms in the context of borderline personality disorder (BPD). Calati et al. [3] administered genetic testing alongside the Temperament and Character Inventory (TCI; [10]) and State Trait Anger Expression Inventory (STAXI; [11]) to community volunteers and a group of consecutively admitted hospital patients representing suicidal and mood-disordered conditions. The study observed that the Met allele corresponded to BPD traits of novelty-seeking, stimulus/sensation traits, harm avoidance, and neuroticism. On the other hand, genotypes without the Met allele had higher pathological aggressiveness and anger. The Met/Met genotype had a strong association with borderline personality pathology. This finding was corroborated by Lazzaretti and colleagues [12], where the Met allele was over-represented in the BPD group compared to those without BPD. These studies follow the findings by Tadic et al. [13], which used structured diagnostic interview data to find connections between BPD symptoms and the Val/Met genotype, despite more BPD patients meeting the Met/Met genotype compared to healthy controls. This extant literature is based on measures of normal personality traits or categorical measures of personality pathology, representing the then-conceptualization of personality and personality pathology.

Our study contributes to the existing literature by investigating how the COMT Val158Met polymorphism associates with self-reported trait-based personality pathology surveyed by an instrument (Personality Inventory for DSM-V (PID-5; [14]) derived from the alternative model of personality disorders (AMPD; [13]). To the best of our knowledge, this study is the first such investigation linking COMT Val158Met polymorphism and dimensional personality pathology, aside from a case example introduced by Madan et al. [15]. As such, this study represents a movement to bridge genetics with an emerging conceptualization of personality pathology. We propose several exploratory hypotheses in the context of the limited current literature. First, we expected personality pathology traits relevant to executive dysfunction and/or emotion dysregulation to be higher among those with at least one Met allele. Additionally, we hypothesized that PID-5 traits relevant to borderline personality pathology (e.g., emotional lability, anxiousness, separation insecurity, depressivity, impulsivity, risk-taking, hostility; [14,15]) would mostly fall under the Met/Met variant.

## 2. Methods

### 2.1. Participants and Setting

Data for this study were collected between January 2018 and October 2024, drawn from individuals with serious mental illness seeking specialized, outpatient assessment and treatment emphasizing personality, genetic, and biological factors (see [16] for the study setup that was integrated with a routine clinical workflow). Eighty-four (*n* = 84) individuals completed genetic testing and measures of dimensional personality pathology. Sample characteristics of the study participants are provided in Table 1.

### 2.2. Measures

Genetic profiles were obtained through Genomind, a DNA-based test analyzing biodata from a cheek swab to identify genotypes of selected polymorphisms relevant to psychiatric medication response. Genomind synthesizes biodata to determine how the body may respond to various medications to facilitate more effective psychopharmacological choices. The test identifies genotypes of 26 SNPs, their relation to 14 psychiatric conditions, and potential gene–drug, drug–drug, and drug–drug–gene interactions. From there, recommendations are offered regarding which medications may be best aligned with the person’s genetic profile. Several meta-analyses indicate that Genomind’s genotype profiling, with greater than 99% accuracy and reproducibility in psychiatric samples, can increase medication adherence and response in complex psychiatric conditions [17,18].

Personality pathology was assessed with the Personality Inventory for DSM-5-Adult (PID-5 [19]) questionnaire, a 220-item self-report instrument assessing functioning as conceptualized by the AMPD, a contemporary dimensional model of personality pathology [20]. Each PID-5 item asks participants to respond to statements about themselves (e.g., I avoid risky situations; I don’t get emotional; I like standing out in a crowd) with a score of either 0 (very false or often false), 1 (sometimes or somewhat false), 2 (sometimes or somewhat true), or 3 (very true or often true). Composite scores from weighted summing provide 25 lower-order trait facet scales and five higher-order trait domain scales representing maladaptive stylistic dispositions. For example, deceitfulness, grandiosity, and manipulativeness are three trait facets subsumed in the trait domain of antagonism, whereas anxiousness, separation insecurity, and emotional lability are the trait facets loading onto the negative affect trait domain. The PID-5 has demonstrated psychometric adequacy across clinical and non-clinical samples [14,21,22].

### 2.3. Procedures

Individuals completed all testing in an outpatient setting. The team psychologist administered all personality inventories in-office or assigned them for at-home completion. All questionnaires were reviewed by the participant and team psychologist during the evaluation for accuracy, reliability, and validity to identify the individual’s test-taking approach. The PID-5 inventories included in this sample represent valid and scorable protocols. The team psychiatrist ordered genetic testing in the first week. Data were collected as part of a larger study examining biomarkers of psychiatric illness and response to treatment.

### 2.4. Data Analyses

The statistical analyses of this study included the non-parametric Kruskal–Wallis H-test to assess group differences in personality pathology among the three genotypes of the COMT Val158Met polymorphism. To correct for the potential of alpha inflation, Bonferroni corrections were applied for inter-group comparisons, with pairwise comparisons performed with the non-parametric Mann–Whitney U test. Non-parametric tests were used throughout for methodological consistency, as most personality measures obtained in our study violated normality assumptions (Shapiro–Wilk’s *p* < 0.05). Statistical significance was set at *p* = 0.05. Sex was considered a potential factor affecting the gene–personality pathology associations, given previous observations on sexually dimorphic effects of the COMT polymorphisms [23,24]. The effect of sex was evaluated with a Mann–Whitney U for differences between males/females in personality pathology trait/domain scores. We employed a stratified analysis (by sex) to examine the joint effect of sex and the COMT polymorphism for group differences in personality traits/domains across the COMT genotypes. Additionally, ordinary least squares regression modeling of traits/domains scores was pursued with COMT genotype, sex, and their interactions as predictors. The models were developed using the statsmodels library [25] (v0.14.4) in Python (v3.13.5, Python Software Foundation 2025).

## 3. Results

### 3.1. Pathological Personality Traits Across Gentopyes of the Val158Met COMT Polymorphism

#### 3.1.1. Broader Domain Scores

**Figure 1:**
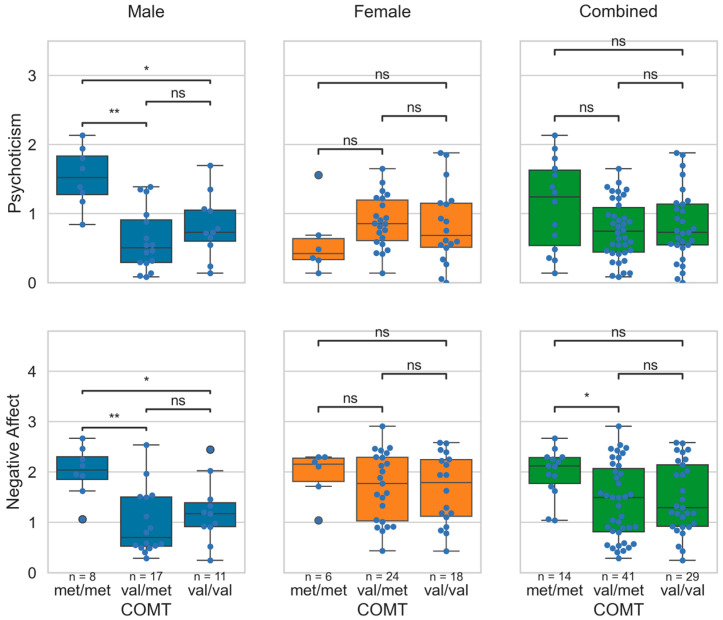
Distribution of the psychoticism and the negative affect scores across the COMT Val158Met polymorphism genotype groups in the male, female, and the combined cohort. In the figure, *: *p* < 0.05; **: *p* < 0.01; *ns*: non-significant. The *p*-values were obtained with Bonferroni corrections applied for multiple group comparisons.

Negative affect was significantly different between the COMT polymorphism groups (H_(2)_ = 6.46, *p* = 0.0396). The Met/Met group (M = 1.98; SD = 0.46) scored higher than the Val/Met group (M = 1.41; SD = 0.74). However, substantial sex-dependent differences across groups were observed. As shown in Figure 1, males with the Met/Met genotype had significantly higher psychoticism and negative affect scores compared to the Val/Met and Val/Val groups. There were no significant differences in any domain scores across the COMT Val158Met polymorphism genotype groups among females.

#### 3.1.2. Pathological Personality Traits

Three of the 25 PID-5 facet traits significantly differed between the COMT Val158Met polymorphism genotype groups in the full cohort. These traits were perceptual dysregulation (H_(2)_ = 7.20, *p* = 0.0273), restricted affectivity (H_(2)_ = 9.04, *p* = 0.0109), and separation anxiety (H_(2)_ = 6.24, *p* = 0.0441). In the perceptual dysregulation scores, for example, the Met/Met group (M = 1.25; SD = 0.60) scored significantly higher than the Val/Met (M = 0.73; SD = 0.47) and the Val/Val group (M = 0.76; SD = 0.53).

In the sex-stratified analysis, the male cohort had nine traits significantly different across the COMT Val158Met polymorphism genotype groups. For example, the unusual beliefs and experiences trait scores were significantly higher in the Met/Met group (M = 0.82; SD = 0.74) compared to the Val/Met (M = 0.61; SD = 0.53) and the Val/Val (M = 0.50; SD = 0.43) group (H_(2)_ = 10.75, *p* = 0.0046). Other traits that were significantly different across the genotype groups were anxiousness, depressivity, distractibility, eccentricity, perceptual dysregulation, risk-taking, separation insecurity, and restricted affectivity. However, there were no significant differences across the genotype groups in any of the 25 traits in the female cohort. The scores across COMT Val158Met genotype groupings for all domains and traits are provided in Table A1 in the Appendix A.

### 3.2. Personality Measures Across Sex

**Figure 2:**
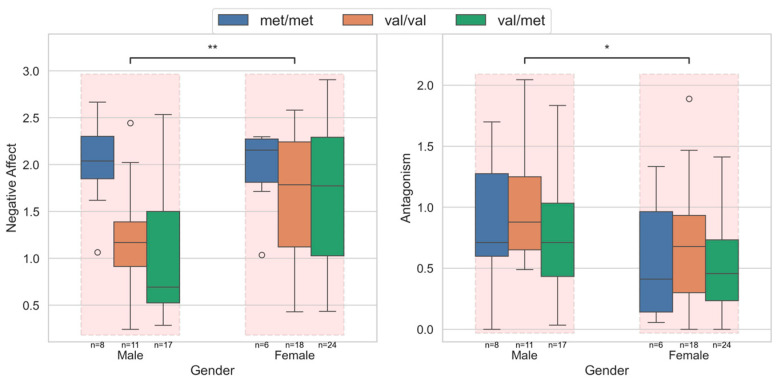
Personality domain scores across sexes for the significantly different negative affect and antagonism domains. The within-group variations across COMT Val158Met polymorphism genotypes are also shown. Males had lower negative affect, primarily driven by the low affect in the Val/Met and Val/Val groups. The antagonism was higher in the male group, with no significant difference associated with the genotypes in either gender. In the figure, *: *p* < 0.05; **: *p* < 0.01.

Group differences in domains and trait scores across sex revealed significant differences in two domains: negative affect and antagonism, and 4 of the 25 facet traits. Females had significantly higher negative affect and lower antagonism than males. Figure 2 shows the distribution of negative affect and antagonism in the male and female cohorts, stratified across the COMT Val158Met polymorphism genotypes. Compared to the females, the lower negative affect in males was primarily due to the lower scores in the Val/Met and Val/Val groups.

Among facet traits, anxiousness and emotional lability (of the negative affect domain), and deceitfulness and grandiosity (of the antagonism domain) traits were significantly different between the sexes.

### 3.3. Gene–Sex Interactions in Dimensional Personality Measures

To validate the above findings of sexual dimorphism effects across COMT genotypes, we followed up with linear regression analyses of the PID-5 domains and facet trait scores with COMT polymorphism genotypes, sex, and their interactions as predictors. The result obtained is shown in Table 2. The modeling that included interactions led to substantial improvement in the adjusted R-squared of the model across multiple domains and traits. Outcomes such as the psychoticism domain score and unusual beliefs and experiences trait score had marked improvements with the inclusion of the interaction term. The R-squared improved from 0.03 to 0.19 for psychoticism score and 0.01 to 0.14 for unusual beliefs and experiences score. The modeling for negative affect, anxiousness, eccentricity, and perceptual dysregulation also improved with the inclusion of the gene–sex interaction.

## 4. Discussion

This study offers preliminary support linking the COMT Val158Met polymorphism and personality pathology as represented in the AMPD. Consistent with our initial hypothesis, the COMT Val158Met polymorphism genotype of Met/Met alleles evidenced higher scores than Val/Met and Val/Val alleles on negative affect, a core domain of personality pathology [3,8]. The Met/Met genotype reflects reduced breakdown of dopamine and norepinephrine, resulting in greater bioavailability of these neurotransmitters and potentially contributing to reduced cognitive and emotional flexibility. Our finding that Met-dominant carriers evidenced higher negative affectivity scores is consistent with prior studies exploring personality and COMT [12,13]. Collectively, the results indicate individuals with Met-dominant genetic penetration are prone to unpleasant emotions that are, in part, related to under-metabolized dopamine and norepinephrine. Interestingly, in the sex-stratified analysis, the elevated negative affect for the Met/Met carriers was significantly higher in males only. This sexual dimorphism was unlikely an effect of statistical power as there were more females than males in our study. Sex-difference findings from our study are consistent with Chen et al.’s [23] recent findings; however, their study reported higher negative emotion for Val-dominant carriers. The discrepancy of our findings with this early study are likely a consequence of differences in the sample population (psychiatric population in our study vs. healthy controls only in Chen et al.’s study [23]) and/or the measurement instruments employed (PID-5 capturing the pathological personality traits in our study vs. a custom negative emotionality constructed from multiple instruments in Chen et al.’s study [23]).

The second hypothesis of this study was partially affirmed, such that Met/Met carriers had higher scores than Val/Met and Val/Val counterparts on traits relevant to borderline personality pathology as proposed by Fowler and colleagues [14,15]. Met/Met individuals scored significantly higher on separation insecurity and marginally higher on depressivity and emotional lability (*p* = 0.0551 and *p* = 0.0577, respectively). The elevated borderline personality pathology-related traits were more profound when only the male cohort was considered. Of the seven BPD-related traits, four facet traits (anxiousness, separation insecurity, depressivity, and risk-taking) were significantly higher in the Met/Met males compared to the Val/Met and Val/Val males. This finding suggests that distinct traits notable in borderline personality pathology correspond with Met/Met carriers compared with other COMT Val158Met polymorphism genotypes. Sexual dimorphism of Met/Met carriers expressing as higher borderline personality pathology-related traits, as observed in our study, is in line with the previously reported sexually dimorphic effect of COMT functional polymorphisms on psychiatric disorders [24] and sexually dimorphic genetic architecture of quantitative traits in general [26]. More broadly, our results uphold a relationship between the Met/Met genotype, negative affectivity, and borderline personality pathology [12,13], if not more general aspects of psychopathology [27]. The PID-5 facet trait of impulsivity, purportedly loading onto the borderline personality pathology scale, did not demonstrate statistically significant differences between the COMT Val158Met genotypes. This may suggest that some traits are salient to COMT and the Met/Met genotype, possibly in a sexually dimorphic fashion. In contrast, other aspects of borderline personality pathology, such as impulsivity, may be less relevant. Given these results, we speculate that greater depressivity and perceptual dysregulation scores observed in Met/Met groups in our study may suggest a vulnerability to depressive realism [28], such that this genotype may underpin characterologically inflexible depressive mindsets (i.e., “My life will never improve; I’m just a burden to everyone”).

In addition, this study observed relationships between the Val/Val genotype and personality pathology traits. The Val/Val genotype reflects an overexpression of the enzyme that breaks down dopamine and norepinephrine, thus reducing the bioavailability of these neurotransmitters. In this study, Val/Val carriers had higher scores than Val/Met carriers on the PID-5 facet trait of restricted affectivity. On one hand, this may suggest that Val-dominant carriers are prone to actively restraining or managing emotions via suppression. On the other hand, this may reflect that Val/Val carriers have lower prefrontal cortex dopaminergic signaling, thus reducing their natural affective responsivity [29,30].

Despite being among the first studies to examine relationships between genetic markers of neurotransmitter activity and dimensional measures of personality pathology, limitations must be acknowledged. Foremost, this study is a candidate gene study that does not comprehensively evaluate genome-wide associations, though the gene X sex analyses did explain a meaningful portion of the variance in one particular domain. While some argue that candidate gene studies are obsolete [31], we contend this study represents a preliminary empirical effort to link genetics with an emerging paradigm shift in psychiatry and future directions might explore associations via a genome-wide association study. Another limitation of our study stems from the categorical genetic mapping of the Genomind system based on whether a genotype is present in an individual or not. Thus, there are no quantitative markers for the degree of COMT polymorphism penetrance. These limitations are potentially compounded by the limited sample size of our study. Typical genetic studies may include hundreds of thousands of participants [32]. Further, limited genetic ancestry and ethnicity of the sample cautions against the generalizability of the findings. Indeed, significant ethnic differences in COMT gene variants have been observed that, when considered alongside sociocultural pressures, may mitigate personality trait expression [33,34].

## 5. Conclusions

Our study is a preliminary attempt to link genetics with a model of personality pathology, emphasizing dimensionality, in contrast to categorical approaches. As such, this study represents an effort to link genetics with an emerging, contemporary model of psychological dysfunction. Furthermore, this study supports a larger movement towards more personalized medicine such that awareness of enzymatic activity in the brain can inform care for those with marked personality pathology.

## Figures and Tables

**Table 1:** Sample characteristics of the study participants.

Characteristics	Value
Number of participants	84
Age (years), mean (SD)	32.73 (13.74)
Sex	36 (42.8%) Males, 48 (57.2%) Females
*Race*	
Caucasian	52 (61.9%)
Non-Caucasian	32 (38.1%)
*Marital status*	
Never married	46 (54.8%)
Currently married	17 (20.2%)
Other/Not reported	21 (25.0%)
*Years of education*	
College or more	52 (61.9%)
High school or lower	18 (21.4%)
Not reported	14 (16.7%)

**Table 2:** Linear regression modeling of PID-5 domains and trait scores using the sex and the COMT Val158Met polymorphism genotypes as predictors, with and without modeling the interaction between these predictors. Only the regression results for those domains/traits with significant results in either modeling approach are shown for brevity.

Outcome	with Interaction		Without Interaction	
	R-Squared (Adjusted)	*p*-Value	R-Squared (Adjusted)	*p*-Value
Domains: Negative Affect ^1^	0.19	0.000871	0.17	0.000599
Psychoticism ^1^	0.19	0.000768	0.03	0.148995
Traits: Anhedonia ^2^	0.05	0.098105	0.07	0.037929
Anxiousness ^2^	0.19	0.000639	0.18	0.000315
Depressivity ^2^	0.12	0.010187	0.14	0.009326
Eccentricity ^2^	0.10	0.020008	0.00	0.398169
Emotional Lability ^2^	0.13	0.008217	0.14	0.001815
Perceptual Dysregulation ^2^	0.17	0.001652	0.09	0.013680
Restricted Affect ^2^	0.14	0.004929	0.14	0.002128
Separation Insecurity ^2^	0.14	0.005051	0.05	0.072751
Unusual Beliefs and Experiences ^2^	0.14	0.004763	0.01	0.325413

^1^ = PID-5 personality trait domain. ^2^ = PID-5 personality trait facet.

## Data Availability

The datasets presented in this article are not made publicly available because of the sensitive nature of biobehavioral data. Requests to access the datasets should be directed to the corresponding author.

## Appendix A

**Table A1:** Mean ± Standard Deviation of the PID-5 personality domain and trait scores across the Met/Met (M/M), Val/Met (V/M), and Val/Val (V/V) groups in different cohorts (all, males, females). The F-stat reports the ANOVA-based F-statistics for inter-group differences and the associated *p*-value.

Cohort	All				Male				Female			
Outcome	M/M	V/M	V/V	H-Stat	M/M	V/M	V/V	H-Stat	M/M	V/M	V/V	H-Stat
*Domain*												
**Antagonism**	0.75 ± 0.55	0.64 ± 0.45	0.79 ± 0.51	1.91 *p* = 0.3849	0.88 ± 0.55	0.76 ± 0.47	0.99 ± 0.45	2.14 *p* = 0.3433	0.57 ± 0.49	0.55 ± 0.41	0.66 ± 0.51	0.44 *p* = 0.8022
**Detachment**	1.28 ± 0.56	1.09 ± 0.63	1.13 ± 0.55	1.11 *p* = 0.5728	1.24 ± 0.62	0.90 ± 0.54	0.98 ± 0.48	1.39 *p* = 0.4988	1.34 ± 0.46	1.22 ± 0.65	1.21 ± 0.58	0.24 *p* = 0.8872
**Disinhibition**	1.37 ± 0.56	1.11 ± 0.56	1.31 ± 0.56	2.92 *p* = 0.2326	1.48 ± 0.45	0.95 ± 0.51	1.29 ± 0.55	4.71 *p* = 0.0950	1.25 ± 0.66	1.23 ± 0.56	1.32 ± 0.56	0.44 *p* = 0.8043
**Negative** **Affect**	1.98 ± 0.46	1.41 ± 0.74	1.48 ± 0.69	6.46 *p* = 0.0396	2.01 ± 0.47	0.98 ± 0.63	1.20 ± 0.60	10.35 *p* = 0.0057	1.94 ± 0.45	1.70 ± 0.67	1.66 ± 0.68	0.55 *p* = 0.7584
**Psychoticism**	1.13 ± 0.63	0.77 ± 0.42	0.82 ± 0.50	3.26 *p* = 0.1957	1.53 ± 0.40	0.61 ± 0.43	0.81 ± 0.44	12.78 *p* = 0.0017	0.59 ± 0.46	0.88 ± 0.37	0.82 ± 0.54	3.00 *p* = 0.2230
*Traits*												
**Anhedonia**	1.88 ± 0.68	1.38 ± 0.80	1.53 ± 0.76	4.33 *p* = 0.1148	1.84 ± 0.75	1.08 ± 0.71	1.38 ± 0.75	4.29 *p* = 0.1168	1.94 ± 0.56	1.60 ± 0.79	1.62 ± 0.75	0.72 *p* = 0.6989
**Anxiousness**	2.14 ± 0.68	1.68 ± 0.91	1.66 ± 0.80	3.26 *p* = 0.1958	2.04 ± 0.65	1.10 ± 0.76	1.33 ± 0.55	7.88 *p* = 0.0195	2.28 ± 0.70	2.10 ± 0.75	1.86 ± 0.86	1.36 *p* = 0.5059
**Attention** **Seeking**	1.26 ± 0.80	1.03 ± 0.76	1.11 ± 0.79	0.62 *p* = 0.7324	1.28 ± 0.66	1.02 ± 0.65	1.53 ± 0.73	3.78 *p* = 0.1509	1.23 ± 0.95	1.04 ± 0.83	0.85 ± 0.71	0.59 *p* = 0.7459
**Callousness**	0.66 ± 0.72	0.31 ± 0.38	0.49 ± 0.41	4.59 *p* = 0.1005	0.74 ± 0.64	0.37 ± 0.50	0.51 ± 0.39	2.95 *p* = 0.2288	0.56 ± 0.79	0.27 ± 0.26	0.48 ± 0.43	1.77 *p* = 0.4129
**Deceitfulness**	0.84 ± 0.66	0.68 ± 0.49	0.74 ± 0.55	0.46 *p* = 0.7939	1.11 ± 0.63	0.75 ± 0.49	0.95 ± 0.45	2.97 *p* = 0.2265	0.48 ± 0.52	0.62 ± 0.47	0.60 ± 0.57	0.69 *p* = 0.7075
**Depressivity**	1.75 ± 0.79	1.13 ± 0.75	1.21 ± 0.72	5.80 *p* = 0.0551	1.81 ± 0.73	0.73 ± 0.59	1.08 ± 0.59	9.20 *p* = 0.0101	1.67 ± 0.86	1.41 ± 0.72	1.29 ± 0.78	0.92 *p* = 0.6322
**Distractibility**	1.97 ± 0.63	1.55 ± 0.77	1.75 ± 0.71	2.99 *p* = 0.2246	2.14 ± 0.68	1.31 ± 0.78	1.79 ± 0.67	6.47 *p* = 0.0393	1.74 ± 0.46	1.72 ± 0.72	1.73 ± 0.74	0.14 *p* = 0.9317
**Eccentricity**	1.31 ± 0.85	0.99 ± 0.64	1.20 ± 0.80	1.60 *p* = 0.4497	1.86 ± 0.57	0.87 ± 0.71	1.24 ± 0.73	8.23 *p* = 0.0163	0.58 ± 0.58	1.08 ± 0.58	1.18 ± 0.84	2.64 *p* = 0.2665
**Emotional** **Lability**	2.16 ± 0.75	1.52 ± 0.92	1.43 ± 0.83	5.70 *p* = 0.0577	1.93 ± 0.55	1.10 ± 0.85	1.22 ± 0.78	5.67 *p* = 0.0586	2.48 ± 0.86	1.81 ± 0.85	1.56 ± 0.83	3.44 *p* = 0.1787
**Grandiosity**	0.40 ± 0.45	0.50 ± 0.60	0.60 ± 0.51	2.39 *p* = 0.3029	0.54 ± 0.53	0.65 ± 0.72	0.83 ± 0.46	2.83 *p* = 0.2424	0.22 ± 0.21	0.38 ± 0.48	0.45 ± 0.48	0.58 *p* = 0.7500
**Hostility**	1.38 ± 0.83	1.00 ± 0.64	1.20 ± 0.67	3.57 *p* = 0.1680	1.54 ± 0.71	0.82 ± 0.59	1.12 ± 0.59	5.92 *p* = 0.0518	1.17 ± 0.93	1.13 ± 0.65	1.24 ± 0.70	0.86 *p* = 0.6501
**Impulsivity**	1.23 ± 0.83	1.03 ± 0.79	1.19 ± 0.74	1.20 *p* = 0.5497	1.38 ± 0.60	0.75 ± 0.57	1.15 ± 0.72	5.05 *p* = 0.0799	1.03 ± 1.03	1.23 ± 0.87	1.22 ± 0.76	0.23 *p* = 0.8901
**Intimacy** **Avoidance**	0.83 ± 0.62	0.74 ± 0.73	0.73 ± 0.58	0.64 *p* = 0.7256	0.62 ± 0.44	0.61 ± 0.53	0.70 ± 0.54	0.25 *p* = 0.8835	1.11 ± 0.72	0.83 ± 0.82	0.75 ± 0.60	0.92 *p* = 0.6302
**Irresponsibility**	0.93 ± 0.69	0.72 ± 0.49	0.92 ± 0.53	2.33 *p* = 0.3113	0.90 ± 0.69	0.70 ± 0.43	0.91 ± 0.44	1.11 *p* = 0.5738	0.98 ± 0.68	0.73 ± 0.52	0.92 ± 0.57	1.27 *p* = 0.5301
**Manipulativeness**	1.00 ± 0.86	0.76 ± 0.65	1.00 ± 0.63	2.74 *p* = 0.2535	1.00 ± 0.79	0.89 ± 0.74	1.18 ± 0.59	1.90 *p* = 0.3858	1.00 ± 0.94	0.65 ± 0.55	0.89 ± 0.63	1.37 *p* = 0.5038
**Perceptual** **Dysregulation**	1.25 ± 0.58	0.73 ± 0.46	0.76 ± 0.52	7.20 *p* = 0.0273	1.54 ± 0.47	0.58 ± 0.45	0.73 ± 0.51	11.68 *p* = 0.0029	0.86 ± 0.47	0.84 ± 0.44	0.78 ± 0.52	0.64 *p* = 0.7274
**Perseveration**	1.63 ± 0.57	1.26 ± 0.72	1.43 ± 0.53	4.45 *p* = 0.1080	1.74 ± 0.44	1.11 ± 0.75	1.43 ± 0.59	4.47 *p* = 0.1068	1.48 ± 0.69	1.37 ± 0.66	1.43 ± 0.49	0.40 *p* = 0.8167
**Rigid** **Perfectionism**	1.34 ± 0.75	1.09 ± 0.78	1.06 ± 0.65	1.42 *p* = 0.4919	1.40 ± 0.64	0.98 ± 0.79	0.86 ± 0.47	2.41 *p* = 0.2996	1.27 ± 0.85	1.17 ± 0.77	1.18 ± 0.71	0.06 *p* = 0.9687
**Risk-taking**	1.51 ± 0.82	1.21 ± 0.71	1.40 ± 0.60	2.73 *p* = 0.2551	1.84 ± 0.52	1.10 ± 0.47	1.44 ± 0.38	8.54 *p* = 0.0140	1.07 ± 0.93	1.30 ± 0.84	1.37 ± 0.70	1.29 *p* = 0.5240
**Separation** **Insecurity**	1.63 ± 0.79	1.06 ± 0.77	1.35 ± 0.72	6.24 *p* = 0.0441	2.05 ± 0.57	0.87 ± 0.64	1.04 ± 0.59	11.24 *p* = 0.0036	1.07 ± 0.69	1.20 ± 0.83	1.55 ± 0.73	2.90 *p* = 0.2349
**Submissiveness**	1.20 ± 0.50	1.54 ± 0.67	1.50 ± 0.67	2.93 *p* = 0.2308	1.31 ± 0.39	1.47 ± 0.55	1.50 ± 0.38	1.21 *p* = 0.5447	1.04 ± 0.58	1.58 ± 0.74	1.50 ± 0.79	2.12 *p* = 0.3471
**Restricted affectivity**	0.87 ± 0.80	0.81 ± 0.63	1.28 ± 0.66	9.04 *p* = 0.0109	0.95 ± 0.89	0.97 ± 0.76	1.74 ± 0.50	7.15 *p* = 0.0280	0.76 ± 0.65	0.70 ± 0.48	1.02 ± 0.59	3.13 *p* = 0.2089
**Suspiciousness**	1.13 ± 0.57	1.11 ± 0.72	1.01 ± 0.52	0.67 *p* = 0.7137	1.34 ± 0.61	0.88 ± 0.57	0.94 ± 0.56	3.87 *p* = 0.1441	0.86 ± 0.37	1.27 ± 0.76	1.05 ± 0.48	1.31 *p* = 0.5191
**Unusual** **Beliefs and Exp**	0.82 ± 0.71	0.61 ± 0.53	0.50 ± 0.42	1.45 *p* = 0.4834	1.19 ± 0.61	0.40 ± 0.33	0.47 ± 0.26	10.75 *p* = 0.0046	0.33 ± 0.52	0.75 ± 0.59	0.51 ± 0.49	3.84 *p* = 0.1469
**Withdrawal**	1.14 ± 0.92	1.15 ± 0.72	1.16 ± 0.64	0.15 *p* = 0.9295	1.25 ± 1.03	0.98 ± 0.71	1.04 ± 0.60	0.17 *p* = 0.9171	0.98 ± 0.72	1.27 ± 0.71	1.23 ± 0.65	0.74 *p* = 0.6905
